# Design of a multi-epitopic vaccine against Epstein-Barr virus *via* computer-based methods

**DOI:** 10.3389/fimmu.2023.1115345

**Published:** 2023-03-14

**Authors:** Amirhossein Larijani, Ali Kia-Karimi, Davoud Roostaei

**Affiliations:** ^1^ Student Research Committee, School of Medicine, Guilan University of Medical Sciences, Rasht, Iran; ^2^ Student Research Committee, School of Medicine, Mazandaran University of Medical Sciences, Sari, Iran; ^3^ Department of Pharmacology, School of Medicine, Guilan University of Medical Sciences, Rasht, Iran

**Keywords:** EBV, multi-epitope vaccine, structural proteins, humoral immunity, cellular immunity, adjuvant

## Abstract

**Background:**

Scientific findings have shown that Epstein-Barr virus (EBV) plays a key role in the development of some tumor diseases. Therefore, this study intends to take a practical step in controlling the pathogenicity of this virus by designing an effective vaccine based on the virus Capsid Envelope and Epstein–Barr nuclear immunogen (EBNA) Proteins Epitopes. Currently, there are no effective drugs or vaccines to treat or prevent EBV infection. So, we applied a computer-based strategy to design an epitope vaccine

**Results:**

We designed a powerful multi-epitope peptide vaccine against EBV using in silico analysis. The vaccine is made up of 844 amino acids derived from three different types of proteins (Envelope, Capsid, EBNA) found in two different viral strains. responses. These epitopes have a high immunogenic capacity and are not likely to cause allergies. To enhance the vaccine immunogenicity, we used rOv-ASP-1, a recombinant Onchocerca volvulus activation associated protein-1, as an adjuvant and linked it to the vaccine’s N and C terminus. The physicochemical and immunological properties of the vaccine structure were evaluated. The proposed vaccine was stable, with a stability index of 33.57 and a pI of 10.10, according to bioinformatic predictions. Docking analysis revealed that the vaccine protein binds correctly with immunological receptors.

**Conclusion:**

Our results demonstrated that the multi-epitope vaccine might be potentially immunogenic and induce humoral and cellular immune responses against EBV. This vaccine can interact appropriately with immunological receptors Also, it has a high-quality structure and suitable characteristics such as high stability.

## 1 Introduction

Epstein–Barr virus (EBV) is a member of the herpes virus family known as Human herpesvirus 4 in scientific sources (1). In 1964, this new herpes virus family was detected, and discovery was made by deriving the virus from tumor cells in experimental culture (2). Human herpesvirus 4 (HHV-4) is a linear double-stranded DNA virus of about 170 kb in length (3).

EBV is further classified into two primary subgroups (type 1 and type 2); these two groups differ mainly in the EBNA-3 (nuclear immunogen-3 gene). Both subgroups are reported worldwide; however, in most of the populations, type 1 is dominant. Sore throat, swelling, fatigue, fever, swollen lymph nodes, and rashes on the skin are the clinical symptoms of infections with EBV.

The virus is transmitted orally and is usually transmitted through contact with close family in childhood and infancy (4). Among the ways to prevent contracting the virus is to reduce close contact with people suspected or infected with the virus and avoiding using an ordinary toothbrush, sharing food, or exchanging bodily fluids (5).

Immunity against EBV has been studied extensively. Natural killer (NK) cells play an important role in the innate immune response, delaying or preventing the EBV transformation of B cells through the production of interferon gamma (IFN-*γ*). Subsequently, the virus elicits strong adaptive immune responses, primarily mediated by cytotoxic CD8 T cells. CD8 T cell responses eliminate viral-infected cells upon recognition of EBV peptide antigens bound to MHC I molecules in the surface of target cells.

Although it affects about 90% of adults worldwide, inactive latent EBV usually does not cause severe health problems. In contrast, the virus can cause diseases such as Burkitt’s lymphoma (BL), Hodgkin lymphoma (HL), nasopharyngeal carcinoma and gastric cancer (6).

There is currently no pharmacological cure for EBV, and no preventative or therapeutic vaccinations are available. Several vaccine trials were conducted between 2006 and 2015, including a candidate vaccine in a phase two trial based on the gp-350 protein that decreased the risk of IM, but not the viral infection. This weakness can be attributed to differences in the characteristics of the vaccine such as effectiveness and the duration of protection offered against EBV infection. According to earlier research, most potential vaccines have a short lifetime, meaning that they might delay the infection, but may not be able to prevent long-term increases in infectious mononucleosis (IM) infections.

Despite several years of research by scientists about the virus, there is still no approved vaccine on the global market for the virus. Therefore, designing a prophylactic multi-epitopic vaccine can be a suitable roadmap and provide a new perspective (7).

The mechanism of persistent EBV virus infection is that it infects B cells and downregulates cell surface HLA class I (8).

Human herpesvirus 4 encodes about 80 genes such as Epstein-Barr Nuclear Immunogen 2 (EBNA-2) and latent membrane protein-1 (LMP-1). Both induce the immortalization of infectious cells in a culture dish outside a living organism. Another example is EBNA-1 which allows the virus to self-replicate (3). The virus also has other proteins like Capsid Envelope Large tegument protein etc.

The EBNA capsid envelope, and LMP of virus are our target proteins to check for finding and predicting Epitopes and eventually make an effective vaccine. For this purpose, we have to use the NCBI server for virus assembly and find Capsid Envelope EBNA and LMP proteins. In the following Homolog, allergen toxin proteins and proteins that are not immunogen became removed. After Epitope prediction and removing inappropriate epitopes, the vaccine candidate and its sequence are determined by using the remaining epitopes and adding the appropriate adjuvant. the final stage is monitoring and controlling the vaccine’s Validation and effectiveness. The criteria of an ideal vaccine include creating a good immunity and being available for the people of the community and maintaining the safety of this vaccine during the coming months or years (9).

## 2 Methods

### 2.1 Finding the virus assembly and screening of target protein

We used the server (https://www.ncbi.nlm.nih.gov/ ) part assembly to find virus assembly. We found Human gammaherpesvirus 4 (GenBank sequence: AJ507799.2) as well as human herpesvirus 4 (GenBank sequence: DQ279927.1) and stored the data (two strains). We looked for required proteins called Epstein-Barr Virus nuclear immunogen (EBNA) and LMP in the stored data related to the EBV virus assembly in two strains.

### 2.2 Finding allergen proteins

Allergen proteins were founded from selected proteins of step 1 using the ALGPRED server (http://crdd.osdd.net/raghava/algpred/ ). This web can determine the allergenicity of proteins that use the Readseq program (http://iubio.bio.indiana.edu/soft/molbio/readseq/ ) and various formats such as EMBL, GCG and FASTA can be uploaded to the web to read the sequence. In the next we removed them from selected proteins and saved the reminds Proteins (10).

### 2.3 Finding homologous proteins

We used the Basic Local Alignment Search Tool (BLAST) section of the NCBI server (https://www.ncbi.nlm.nih.gov/) for finding homologous proteins with human and laboratory animal proteins (mice) and extract them from target proteins. BLAST can identify similar local regions of sequences. the program compares the sequences of nucleotides or proteins in the sequence of databases and reports the results. At the end of this section, we saved proteins that weren’t Homolog (11).

### 2.4 Immunogenicity of proteins

Obtain the immunogenicity of each of the selected proteins with the VaxiJen server (http://www.ddg-pharmfac.net/vaxijen/VaxiJen/VaxiJen.html) and save scores. This site uses viral and tumor bacterial datasets (each set 100 known immunogens and 100 non-immunogens) to predict protein immunogenicity. In general, this site uses internal leave-one-out cross-validation and external validation and an additional five training sets for each class of immunogens to determine the final immunogenicity and stability results (12).

### 2.5 Toxicity

Controlling and monitoring the toxicity of proteins and removing toxic proteins was done using Toxinpred serve (https://webs.iiitd.edu.in/raghava/toxinpred/pep_test.php). The tools used in the site in the field of datasets include main datasets and alternative datasets, and in the field of forecasting tools and prediction approaches, it uses the SVM system, in which methods and tools such as 1. Amino Acid Composition, 2. Dipeptide Composition, 3. Binary Profile pattern, 4. Motif-based profiles, and more Hybrid Methods and Quantitative Matrix are used (13).

### 2.6 Epitope prediction

In this step, we predict the epitopes for each selected protein. Nhlapred server (http://crdd.osdd.net/raghava/nhlapred/) was used for MHC1. This site has two major sections called Artificial neural network ANNPred and ComPred that ANNPred was used in this article (14). Also, Mhc2pred server (http://crdd.osdd.net/raghava/mhc2pred/) is used for MHC2. MHC2Pred is SVM based method for MHC class II Binders predictions with 80% accuracy for 42 alleles in the SVM method (15).

The Bcpred server was used for linear B cell epitopes prediction. The present version of BCPREDS allows the user to choose between three prediction methods: (i) AAP implementation [Chen et al., 2007], (ii) BCPred [EL Manzalawy et al., 2008], and (iii) FBCPred [EL Manzalawy et al., 2008b]. Users can submit an immunogen sequence and, if desired, an epitope length and specificity threshold. The results are presented in a variety of ways that are easy to understand (16).

### 2.7 Epitope allergenicity

Select non-Allergen Epitopes by using the Allertop server (http://www.pharmfac.net/allertop/) and remove Allergens one.

Allertop is an allergenicity prediction site based on ACC preprocessing on a set of fabricated allergens and level-independent models used to detect allergens based on chemical properties In this site, allergen sets of food, poison and inhalant have been used, and Allertop is 94% better than other sites it works (17).

### 2.8 Epitops immunogens

Select immunogen and immunogen epitopes using the Immunogenicity prediction server (http://www.ddg-pharmfac.net/vaxijen/VaxiJen/VaxiJen.html) (12).

### 2.9 Epitope toxicity

Identify and remove toxic epitopes using (https://webs.iiitd.edu.in/raghava/toxinpred/pep_test.php) and ultimately select the best Epitope (13).

### 2.10 Creat a vaccine candidate

After predicting the epitopes, sixteen epitopes from two different virus strains were linked to each other with appropriate linkers(KK) and the vaccine candidate was identified.

At this stage, adjuvant (Ov-ASP-1) (18) was added to the beginning and the end of the vaccine by using an appropriate linker(EAAAK).

#### 2.10.1 Investigation of the second structure and measurement of the amount of alpha-helix of the determined vaccine

To determine the second structure And measurement of alpha-helix levels for the determined vaccine, we used the PRABI site and the GORV IV (https://npsa-prabi.ibcp.fr/cgi-bin/npsa_automat.pl?page=/NPSA/npsa_gor4.html) method. In this method, the amount of alpha-helix was measured. The GOR method is based on the information theory that GOR IV uses all possible pair frequences of 17-amino acid windows (19).

#### 2.10.2 Physiochemical assay of the vaccine structure

Use of the Expassy (version of Protoparam software), (https://web.expasy.org/protparam/) to measure the vaccine candidate stability. Protoparam is a tool that calculates the various physical and chemical properties of the protein sequence given to it (20).

### 2.11 Tertiary structure 3D-modeling and validation

#### 2.11.1 Use of Robetta server to receive the PDB format of vaccine candidate protein and its tertiary protein structure.

Robetta(https://robetta.bakerlab.org/submit.php) Sequence analysis and production of structural models using comparative modeling and *De Novo* prediction are used to predict the structure, which can lead to two paths. If the protein matched the known structure using BLAST, PSI-BLAST, FFAS03, or 3D-Jury, it is used as a comparative modeling model, and if it does not match, the prediction is performed using the *de novo* Rosetta fragment insertion method (21).

#### 2.11.2 Refining the predicted model by Robetta, using the Galaxy refine web server

The GalaxyWEB server refines the terminus region or loop of protein structure by *ab initio* modeling. This server is well tested and validated by the CASP10 (Critical assessment of techniques for protein structure prediction) method and it uses gentle and aggressive relaxation methods to refine proteins. Finally, Galaxy Refine showed five structures as modified models of vaccine structure (22).

The procheck server (https://saves.mbi.ucla.edu/) was used to validate the 3D-Structure obtained from the Galaxy refine web server by using the Ramachandran plot and ERRAT number (23).

### 2.12 prediction of discontinuous B-cell epitopes using Ellipro

Using ElliPro from the IEDB database (http://tools.immuneepitope.org/tools/ElliPro), the 3D structure of the designed vaccine to predict the discontinuous B-cell epitopes was examined. Ellipro is an apropreiate tool for predicting dicontinous B-cell epitopes based on implements Thornton’s method and residue clustering algorithm. this tool provides the MODELLER program and the Jmol viewer which can be used to prediction and visualization of epitopes in a given protein sequence or structure (24).

### 2.13 Using HDOCK Server to measure the docking rate of designed vaccine by both types of MHC

HDOCK (http://hdock.phys.hust.edu.cn/) runs protein-protein and protein-DNA/RNA docking and the researcher can enter the sequence and structure of proteins into the site and receive it 10 to 20 minutes after docking. When the best 10 predictions from five docking benchmarks were assessed, the HDOCK pipeline tied with template-based modeling on the protein-protein and protein-DNA benchmarks and performed better than template-based modeling on the three protein-RNA benchmarks (25).

## 3 Results

### 3.1 Screening of potential target proteins

In assembly strain 1 (human gammaherpesvirus 4), we found six capsids, 4 EBNAs, one envelope, and 3 L-amps (**Table 1**). We also found five capsids, 7 EBNAs, six envelopes, and 3 LMPs from strain 2 (human herpesvirus 4) (**Table 2**).

**Table 1:** Selected proteins from an assembly of Human gamma herpes virus4.

Protein name	Protein ID
Capsid protein VP26	CAD53401.1
Capsid assembly protein	CAD53403.1
Putative capsid protein VP19C	CAD53404.1
Putative capsid protein vp23	CAD53446.1
Major capsid protein	CAD53447.1
Capsid protein P40	CAD53454.1
EBNA3A nuclear protein	CAD53419.1
EBNA-3B nuclear protein	CAD53420.1
EBNA3C latent protein	CAD53421.1
EBNA-1 protein	CAD53427.1
Envelope glycoprotein gp350	CAD53417.1
Terminal protein LMP2A	CAD53382.1
Terminal protein LMP2B	CAD53383.1
Latent membrane protein	CAD53472.1

**Table 2:** Selected proteins from an assembly of human herpesvirus 4.

Protein name	Protein ID
small capsid protein on hexon tips	ABB89228.1
capsid triplex protein	ABB89231.1
major capsid protein	ABB89273.1
minor capsid scaffold protein	ABB89280.1
major capsid scaffold protein	ABB89281.1
EBNA-LP	ABB89221.1
EBNA-2	ABB89222.1
EBNA-3A	ABB89243.1
EBNA-3B	ABB89244.1
EBNA-3C	ABB89245.1
EBNA-1	ABB89251.1
EBNA-1 triplet	ABB89252.1
envelope glycoprotein gN	ABB89240.1
envelope glycoprotein gB	ABB89287.1
envelope glycoprotein gH; gp85	ABB89276.1
envelope glycoprotein gM	ABB89259.1
envelope glycoprotein gp42	ABB89246.1
envelope glycoprotein gp350	ABB89242.1
LMP-2A	ABB89217.1
LMP-2B	ABB89219.1
LMP-1	ABB89294.1

### 3.2 Allergenicity

Using the **Algepred** server, we measured the allergenicity of the target potential proteins, which in strain 1 were all non-allergenic capsid and EBNA, LMP, and envelop proteins. In strain 2, one of the EBNA proteins and three of the envelopes were allergenic (**Table 3**). We removed the allergen proteins at this stage, and the non-allergens were taken to the next step for other assays.

**Table 3:** Allergen and non-allergen proteins in strain 2.

Protein name	Allergen	Non-allergen
small capsid protein on hexon tips		✓
capsid triplex protein		✓
major capsid protein		✓
minor capsid scaffold protein		✓
major capsid scaffold protein		✓
EBNA-LP	✓	
EBNA-2		✓
EBNA-3A		✓
EBNA-3B		✓
EBNA-3C		✓
EBNA-1		✓
EBNA-1 triplet		✓
envelope glycoprotein gN	✓	
envelope glycoprotein gB		✓
envelope glycoprotein gH; gp85		✓
envelope glycoprotein gM		✓
envelope glycoprotein gp42	✓	
envelope glycoprotein gp350	✓	
LMP-2A		✓
LMP-2B		✓
LMP-1		✓

### 3.3 Homology

Using the NCBI database and Blastp software, we examined the homology of the proteins selected in the previous step. We concluded that none of them are homologous to human and mouse proteins. The software algorithm at this stage was blast (protein-protein blast).

### 3.4 Immunogenicity

At this stage, we tested the immunogenicity of the proteins using the **VaxiJen** server and changed the default of the threshold of the server, which was 0.45, to a higher digit of 0.47. With this calculation, in strain 1, two capsids and 4 EBNA and one of the LMPs had good immunogenicity, and in strain 2, 4 capsids, 5 EBNA, three envelopes, and 2 LMPs were suitable immunogens (**Tables 4**, **5**).

**Table 4:** Immunogenicity of human gammaherpesvirus 4 (strain 1) proteins.

Protein name	Immunogen	Non-immunogen	Score
capsid protein VP26		**✓**	0.4699
capsid assembly protein		**✓**	0.4676
putative capsid protein VP19C		**✓**	0.4333
putative capsid protein vp23	**✓**		0.5147
major capsid protein		**✓**	0.4286
capsid protein P40	**✓**		0.4765
EBNA3A nuclear protein	**✓**		0.4731
EBNA-3B nuclear protein	**✓**		0.6105
EBNA3C latent protein	**✓**		0.5178
EBNA-1 protein	**✓**		0.5545
envelope glycoprotein gp350	**✓**		0.5756
terminal protein LMP2A	**✓**		0.5051
terminal protein LMP2B	**✓**		0.4915
latent membrane protein		**✓**	0.3362

**Table 5:** Immunogenicity of human herpesvirus 4 (strain 2) proteins.

Protein name	Immunogen	Non-immunogen	score
small capsid protein on hexon tips	✓		0.4724
capsid triplex protein		✓	0.4230
major capsid protein		✓	0.4289
minor capsid scaffold protein	✓		0.4771
major capsid scaffold protein	✓		0.4745
EBNA-2	✓		0.5544
EBNA-3A	✓		0.4877
EBNA-3B	✓		0.5289
EBNA-3C	✓		0.5241
EBNA-1	✓		0.5193
EBNA-1 triplet		✓	0.3411
envelope glycoprotein gB	✓		0.4984
envelope glycoprotein gH; gp85	✓		0.5345
envelope glycoprotein gM	✓		0.4826
LMP-2A	✓		0.4997
LMP-2B	✓		0.4901
LMP-1		✓	0.3870

### 3.5 Toxicity of protein

At this stage, the toxicity of proteins was investigated using the ToxinPred server. Considering that the algorithm of this server toxicity measures proteins in ten amino acid sequences, we selected the best ones in terms of non-toxicity, which is in strain 1 (**Table 6**). Three proteins and in strain 2, 3 proteins remained, and LMP proteins in both strains were removed during the steps.

**Table 6:** Selected non-toxin proteins in both strains.

Proteins name	
capsid protein P40
EBNA3A nuclear protein
envelope glycoprotein gp350
major capsid scaffold protein
EBNA-2
envelope glycoprotein gH; gp85

### 3.6 Epitope prediction

We predicted the epitopes of the virus to B-cell epitopes and MHC1 and MHC2.

#### 3.6.1 Prediction of B-cell epitopes

Using **BCPREDS**: B-cell epitope prediction server, 14 epitopes were identified from capsid proteins in both strains, 19 epitopes from EBNA proteins, and 14 epitopes for envelope proteins in total for both songs.

#### 3.6.2 Prediction of MHC class 1 and MHC class 2 epitopes

In this section, we predicted the epitopes using the **nHLAPred** server for MHC Class 1 and the **MHC2Pred** server for MHC Class 2, and our approach at this stage was that the predicted epitopes in both types of MHC 1 and 2 are the same to stimulate both cellular and humoral immunity well.

#### 3.6.3 Allergenicity of epitopes

At this stage, we examined the allergenicity of the epitopes using the AllerTOP 1.0 server, and some of the epitopes were removed by this method and did not find their way to the next steps.

#### 3.6.4 Immunogenicity of epitopes

Using the **VaxiJen** server, we measured the immunogenicity of epitopes. At this stage, our approach was to select epitopes with the highest immunogenicity score, and from both strains 1 and 2 of the virus, an appropriate number of epitopes were selected from EBNA, Capsid, and envelope proteins.

#### 3.6.5 Choose the best epitope

After checking the toxicity of the epitopes using the ToxinPred server

Finally, the best epitopes were identified as follows: (Immunogenicity rate)

For MHC class 1 and 2, epitopes with more than one immunogenic score were selected. For B cell epitopes in all sections except envelope glycoprotein gp350 and envelope glycoprotein gH; gp85, the top two epitopes were selected in terms of immunogenic score, except three epitopes for gp350 and one epitope for gp85.Finally, with this approach, twelve epitopes were selected as B-cell epitopes and sixteen epitopes for MHC class 1 and 2 (**Table 7**). It should be noted that B-cell epitopes were the same for both capsids, which were considered once in the final candidate for the vaccine.

**Table 7:** selected Epitopes for the vaccine.

Proteins name	B CELL epitopes	Immunogenicity score	MHCs epitopes	Immunogenicity score
capsid protein P40	NLDNKPPRQTPLPYAAPLPP	0/9253	PLPLTVEHL	1/5307
	PPPPPTSHQAAQAQPPPPGT	0/7542	YGTDLAWVL	1/4357
EBNA3A nuclear protein	SDSSSEKEAEDAHLEPAQKG	1/101	SRGGPKVKR	1/6532
	ASMGPVPPVPATQPQYFDIP	1/0598	KDRPGPPAL	1/2528
			AIGAAATRI	1/1735
envelope glycoprotein gp350	TSPTPAGTTSGASPVTPSPS	0/6514	WASLAVLTL	1/02
	TNHTLGGTSPTPVVTSQPKN	0/9229	ITQVTPASI	1/1754
	TAVPTVTSTGGKANSTTGGK	0/7654	LQWASLAVL	1/6217
			TSSKLRPRW	1/7913
major capsid scaffold protein	*PPPPPTSHQAAQAQPPPPGT	0.7542	AQAQPPPPG	1.1051
	*NLDNKPPRQTPLPYAAPLPP	0.9253	RQTPLPYAA	1.4108
EBNA-2	TLQPTPPPRPTLPQPRNQPA	0.6823	GRGRWRGRG	2.6547
	QTPPTNTKQGPDQGQGRGRW	0.8481	TLQPTPPPR	1.5789
			ENVGGPSKR	1.0672
Envelope glycoprotein gH; gp85	DEKEGLETTTYITSQEVQNS	0.6511	SNYFDFDNL	1.1079
			CIFCGFALL	1.0186

### 3.7 Determining the final candidate for the vaccine with the appropriate adjuvant

26 epitopes with high scores were selected to be used in the final vaccine candidate. From sequence 376-226, T-cell and B-cell epitopes related to two envelope proteins from two strains were placed with an appropriate linker. Also, from sequence 379-464, epitopes related to capsid proteins, and from sequence 467-620, epitopes related to EBNA proteins were included with the appropriate linker.

**Figure 1:**
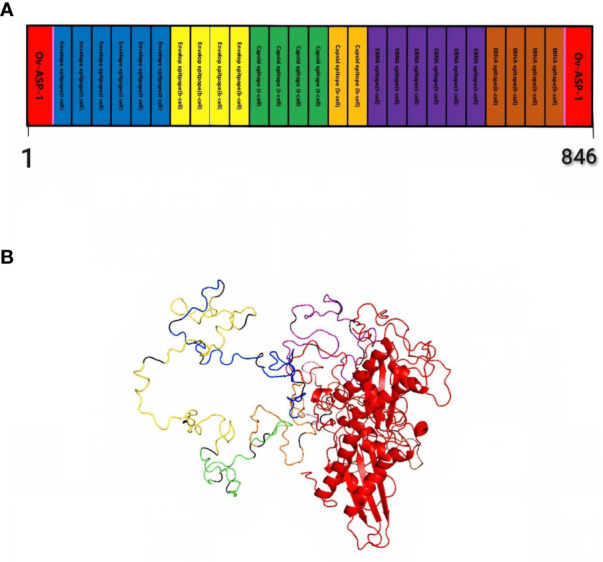
Schematic representation of multi-epitope vaccine. The vaccine consists of 28 sections: epitopes of envelope proteins, capsids, EBNA, and adjuvants: OV-ASP-1 was attached to the beginning and end of the epitopic sequence with a suitable linker **(A)** Tertiary structure of the modeled multi-epitope vaccine construct **(B)** The 3D structure of the vaccine was predicted using a Robetta server model based on homology modeling. The best-predicted model, visualized using PyMOL software. N-and C-terminus of OV-ASP-1 is shown in red, T cell epitopes of envelope proteins in blue, B-cell epitopes of envelope proteins in yellow, T cell epitopes of capsid proteins in green, B-cell epitopes of capsid proteins in orange, T cell epitopes of EBNA proteins in purple, B-cell epitopes of EBNA proteins in brown, EAAAK linkers in pink and KK linkers are shown in black.

Finally, by connecting the epitopes with the appropriate linker and adding the adjuvant OV-ASP-1 to the beginning and end of the epitopic sequence using the appropriate linker, the final candidate for the vaccine was identified. (KK) was used for linking between epitopes and linker (EAAAK) was used to connect adjuvant to the first and last epitope. The final multi-epitope peptide vaccine was 844 amino acid residues (**Figure 1**).

### 3.8 Investigation of the second structure and measurement of the amount of alpha-helix of the determined vaccine

Using the PRABI services and the GORV IV method which it based on the information theory, the second structure of the vaccine and its alpha-helix rate were measured. Accordingly, the alpha-helix rate was reported to be less than 20%, which is acceptable. The result of reviewing other characteristics related to the measured structure is reported in the relevant table (**Table 8**).

**Table 8:** Results of the study of the second structure of the determined vaccine.

Name of the examined unit	Number of residues	Percentage
Alpha helix	135	16%
3_10_ helix	0	0.00%
Pi helix	0	0.00%
Beta bridge	0	0.00%
Extended strand	199	23.58%
Beta turn	0	0.00%
Bend region	0	0.00%
Random coil	510	60.43%
Ambiguous states	0	0.00%
Other states	0	0.00%

### 3.9 Physiochemical assay of the vaccine structure

Using Protoparam software from EXPASY Server, we measured the physicochemical properties of the designed vaccine. The results showed that the molecular weight(MW) and heoretical isoelectric point (pI) of the vaccine protein were 92.51412 kDa and 10.10. The estimated half-life was 30 hours (mammalian reticulocytes, *in vitro*) and 20 hours (yeast, *in vivo*), and 10 hours (Escherichia coli, *in vivo*). The instability index (II) is computed to be 33.57 that showed the vaccine protein was stable (**Table 9**).

**Table 9:** Analysis of physicochemical properties of the EBV vaccine.

Physiochemical properties	value
Instability index	33.57
Aliphatic index	61.73
Grand average of hydropathicity (GRAVY)	-0.745
No. of atoms	13117
Total No. of negatively charged residues (Asp + Glu)	50
Total No. of positively charged residues (Arg + Lys)	147
Molecular weight(Da)	92514.12
Theoretical pI	10.10
No. of amino acids	844

### 3.10 Tertiary structure 3D-modeling and validation

**Figure 2:**
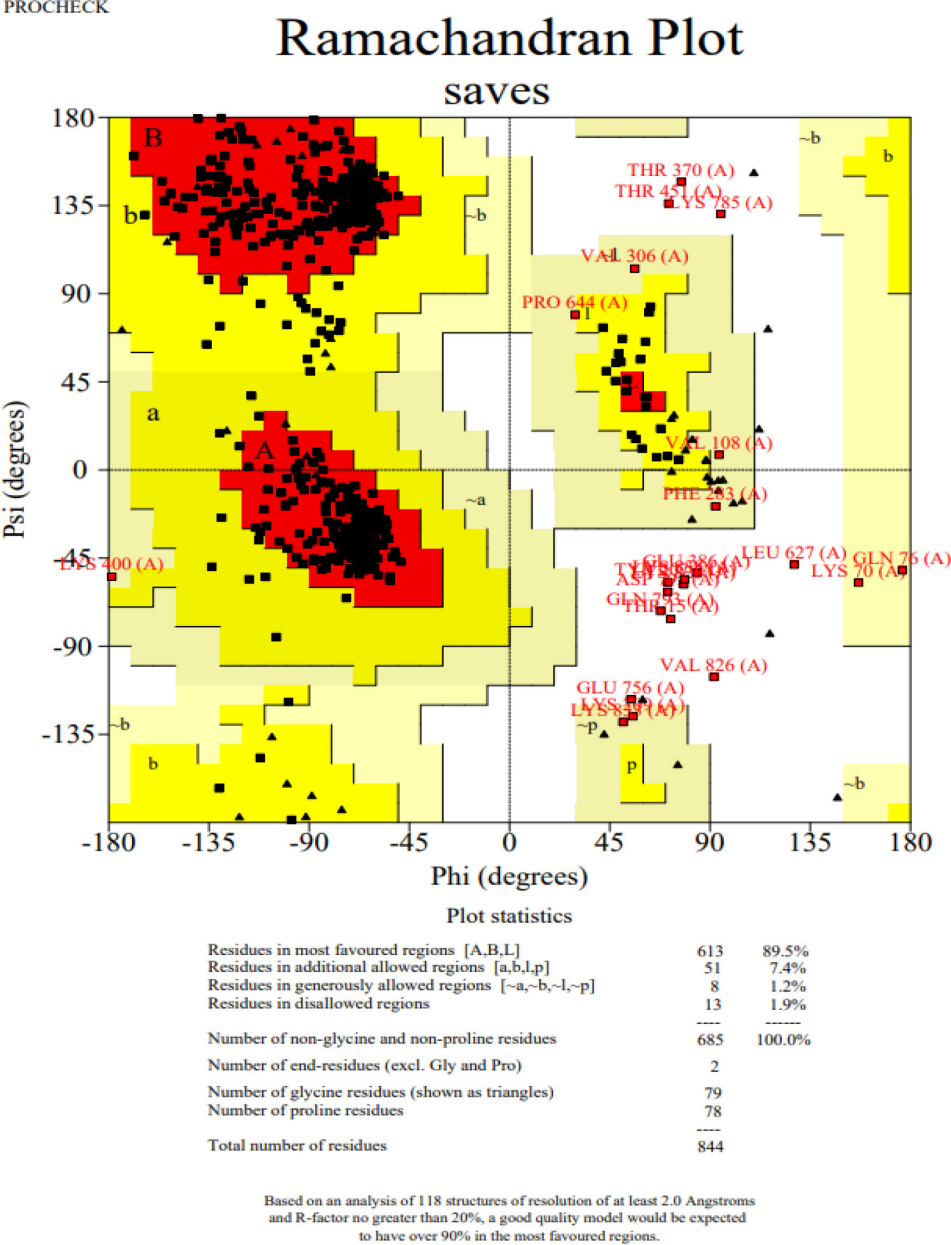
Post-modeling analyses of the 3D structure of VACCINE. Ramachandran plot depicting the stereochemical quality of VACCINE.

**Figure 3:**
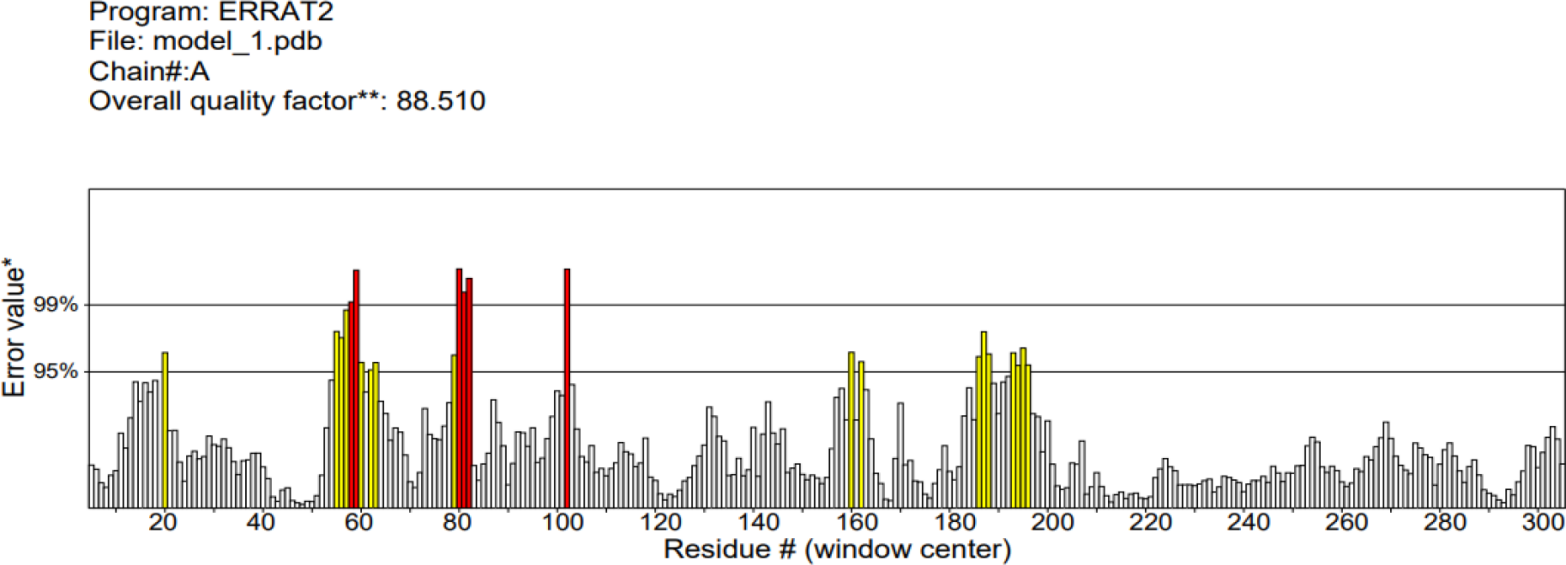
ERRAT diagram depicting the quality of the modeled structure of VACCINE. *On the error axis, two lines are drawn to indicate the confidence with which it is possible to reject regions that exceed that error value.**Expressed as the percentage of the protein for which the calculated error value falls below the 95% rejection limit. Good high resolution structures generally produce values around 95% or higher. For lower resolutions (2.5 to 3A) the average overall quality factor is around 91%. #residue refers to a single unit that makes up a polymer, such as an amino acid in a polypeptide or protein.

The 3D structure of the determined vaccine was determined using the Robetta server based on comparative modeling and *De Novo* prediction and the designated PDB file was received. The quality of the designed vaccine was assessed using the Ramachandran plot in the PROCHECK server and the characteristic atomic interaction in the ERRAT server **(** **Figures 2**, **3** **).** The results of the PROCHECK server showed that the percentage of residues in the most favored regions is 89.5 and the percentage of residues in additional allowed regions is 7.4 and the percentage of residues in generously allowed regions is 1.2 and also the percentage of Residues in disallowed regions is 1.9. The result of the ERRAT server showed that the ERRAT score of this model is 88.510. The outputs obtained from Ramachandran Plot and ERRAT Server showed that the determined model is reliable and usable in other stages. It should be noted that we refined the model predicted by Robetta using the Galaxy refine web server. It then showed five structures as modified models of the vaccine structure, and we used the best model provided by default (**Table 10**).

**Table 10:** structure information of galaxy refine web server.

Model	GDT-HA	RMSD	MolProbity	Clash score	Poor rotamers	Rama favored
Initial	1.0000	0.000	1.431	2.4	0.0	93.7
MODEL 1	0.9639	0.382	1.986	13.8	0.6	95.1
MODEL 2	0.9606	0.389	1.947	12.3	0.6	95.0
MODEL 3	0.9627	0.382	1.984	12.6	0.8	94.5
MODEL 4	0.9630	0.401	1.990	13.5	0.6	94.9
MODEL 5	0.9612	0.399	1.991	12.8	0.6	94.5

### 3.11 Identification of conformational B-cell epitope

Using ElliPro in IEDB, the tertiary designed-vaccine structure was used to predict conformational (discontinuous) B-cell epitope. From 844 amino acid residues, 459 were defined as discontinued B-cell epitope (**Table 11**).

**Table 11:** Information of residues.

No.	Residues	Number of residues	Score
1	A:G509, A:K510, A:K511	3	0.991
2	A:R481, A:P482, A:G483, A:P484, A:P485, A:A486, A:L487, A:K488, A:K489, A:A490, A:I491, A:G492, A:A493, A:A494, A:A495, A:T496, A:R497, A:I498, A:K499, A:K500, A:G501, A:R502, A:G503, A:R504, A:W505, A:R506, A:G507, A:R508, A:T512, A:L513, A:Q514, A:P515, A:T516, A:P517, A:P518, A:P519, A:R520	37	0.958
3	A:R476, A:K477, A:K479, A:D480	4	0.924
4	A:G320, A:T321, A:S322, A:P323, A:T324, A:P325, A:V326, A:V327, A:T328, A:S329, A:Q330, A:P331, A:K332, A:K334, A:K335, A:T336, A:A337, A:V338, A:P339, A:T340, A:V341, A:T342, A:S343, A:T344, A:G345, A:G346, A:K347, A:A348, A:N349, A:S350, A:T351, A:T352, A:G353, A:G354, A:K355, A:K356, A:K357, A:D358, A:E359, A:K360, A:E361, A:G362, A:L363, A:E364, A:T365, A:T366, A:T367, A:Y368, A:I369, A:T370, A:S371, A:Q372, A:E373, A:V374, A:Q375, A:N376, A:S377, A:K378, A:K379, A:P380, A:L381, A:P382, A:L383, A:T384, A:V385, A:E386, A:H387, A:L388, A:K389, A:K390, A:Y391, A:G392, A:T393, A:D394, A:L395, A:A396, A:W397, A:V398, A:L399, A:K401, A:A402, A:Q403, A:A404, A:Q405, A:P406, A:P407, A:P408, A:P409, A:G410, A:K411, A:K412	91	0.827
5	A:L435, A:P436, A:Y437, A:A438, A:A439, A:P440, A:L441, A:P442, A:P443, A:K444, A:K445, A:P446, A:P447, A:P448, A:P449, A:P450, A:T451, A:S452, A:H453, A:Q454, A:A455, A:A456, A:Q457, A:A458, A:Q459, A:P460, A:P461, A:P462, A:P463, A:G464, A:T465, A:K466, A:K467, A:S468, A:R469, A:G470, A:G471, A:P472, A:K473, A:V474, A:K475	41	0.816
6	A:K521, A:K522, A:E523, A:N524, A:V525, A:G526, A:G527, A:P528, A:S529, A:K530, A:R531, A:K532, A:K533, A:S534, A:D535, A:S536, A:S537, A:S538, A:E539, A:K540, A:E541, A:A542, A:E543, A:D544, A:A545, A:H546, A:L547, A:E548, A:P549, A:A550, A:Q551, A:K552, A:G553, A:K554, A:K555, A:A556, A:S557, A:M558, A:G559, A:P560, A:V561, A:P562, A:P563, A:V564, A:P565, A:A566, A:T567, A:Q568, A:P569, A:Q570, A:Y571, A:F572, A:D573, A:I574, A:P575, A:K576, A:K577, A:T578, A:L579	59	0.782
7	A:Q580, A:P581, A:T582, A:P583, A:P584, A:P585, A:R586, A:P587, A:T588, A:L589, A:P590, A:Q591	12	0.617
8	A:N669, A:G670, A:K671, A:L672, A:K673, A:N674, A:R675, A:N676, A:G677, A:T678, A:Y679, A:M680, A:P681, A:R682, A:G683, A:K684, A:N685, A:M686, A:S749, A:K750, A:L751, A:P752, A:K753, A:L754, A:Y755, A:E756, A:N757, A:N758, A:P759, A:S760, A:N761, A:N762, A:M763, A:T764, A:W765, A:K766, A:V767, A:A768, A:G769, A:Q770, A:G771, A:V772, A:L773, A:T776, A:W780, A:G781, A:G809, A:G810, A:N811, A:M812, A:V813, A:G814, A:E815, A:V816, A:I817, A:Y818, A:H819, A:R820, A:G821, A:N822, A:P823, A:C824, A:K825, A:V826, A:D827, A:K828, A:D829, A:C830, A:Y831, A:K834, A:C835, A:G840	72	0.61
9	A:T15, A:G16, A:Y17, A:N18, A:C19, A:P20, A:G21, A:G22, A:K23, A:L24, A:T25, A:A26, A:L27, A:E28, A:R29, A:K30, A:K31, A:I32, A:G34, A:Q35, A:N37, A:K38, A:Y39, A:S41, A:D42, A:I44, A:N45, A:G46, A:K47, A:L48, A:K49, A:N50, A:N52, A:G53, A:T54, A:Y55, A:M56, A:P57, A:R58, A:G59, A:K60, A:M62, A:E64, A:L65, A:W67, A:Q81, A:C82, A:I83, A:A101, A:Y102, A:W103, A:S104, A:S105, A:V106, A:S107, A:V108, A:E109, A:G110, A:L111, A:K112, A:K113, A:T114, A:A115, A:G116, A:T117, A:D118, A:G120, A:K121, A:W124, A:L127, A:P128, A:K129, A:N137, A:V166, A:A167, A:T168, A:Q169, A:C170, A:D171, A:G172, A:G173, A:R174, A:T175, A:L176, A:I177, A:Y194, A:H195, A:R196, A:G197, A:N198, A:P199, A:C200, A:K201, A:V202, A:D203, A:K204, A:D205, A:C206, A:K209, A:K210, A:C211, A:L212, A:S213, A:K214, A:S215, A:G216, A:L217, A:C218, A:K258, A:S261, A:K262, A:L263, A:R264, A:P265, A:R266, A:K268	116	0.581
10	A:Q705, A:C706, A:I707, A:F708, A:G709, A:H710, A:S711, A:P712, A:R713, A:Q714, A:Q715, A:R716, A:E717, A:G718, A:E721, A:S728, A:S729, A:V730, A:Q793, A:C794, A:D795, A:G796, A:G797, A:R798	24	0.534

### 3.12 Identifying protein-protein docking and binding sites

**Figure 4:**
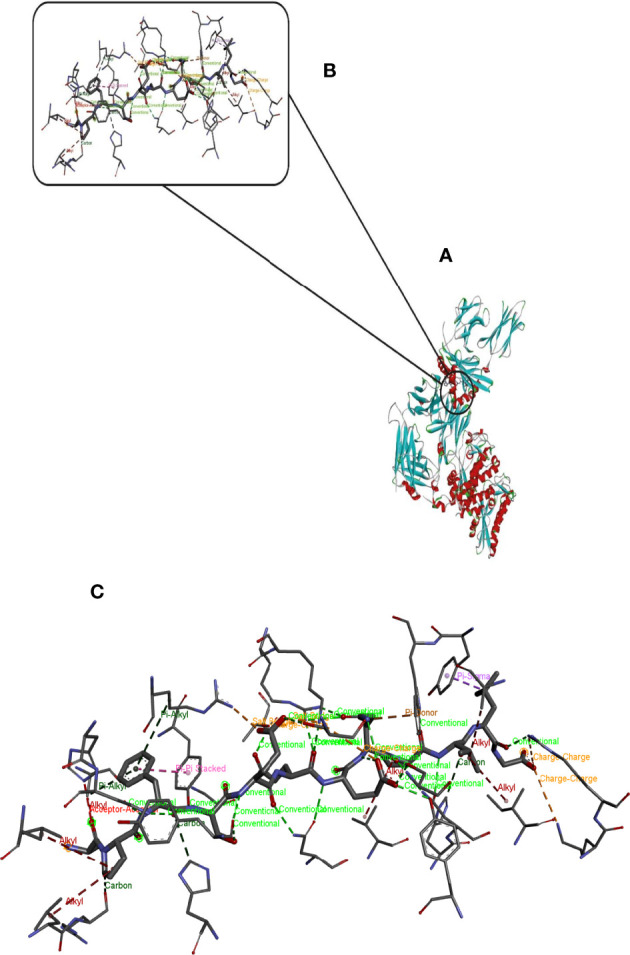
Docking model (cartoon representation) of MHC 1 in complex with the vaccine obtained using HDOCK server. Docked model was visualized via Discovery studio 4.5 software. **(A)** Full view of the docked model **(B)** Magnified view of atomic interactions **(C)** Enlarged view of part **(B)**.

**Figure 5:**
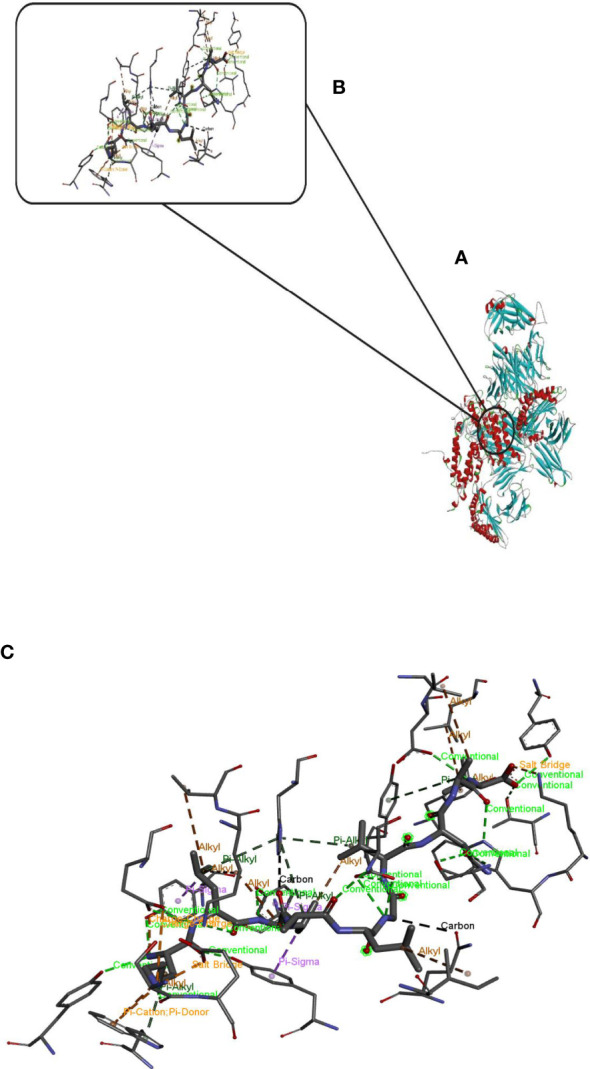
Docking model (cartoon representation) of MHC 2 in complex with the vaccine obtained using HDOCK server. Docked model was visualized via Discovery studio 4.5 software. **(A)** Full view of the docked model **(B)** Magnified view of atomic interactions **(C)** Enlarged view of part **(B)**.

Hdock server has been used to perform the docking of the vaccine with HLA class I histocompatibility immunogen, A-2 alpha chain, and HLA class II histocompatibility immunogen DQ alpha chain molecules. The docked model was selected based on the most ligand rmsd and the best docking score of vaccine and each of the receptors among the 100 models generated by the Hdock for each molecule (**Figures 4**, **5**).

## 4 Discussion

Scientific findings have shown that EBV plays an important role in the development of some tumors. EBV infection is associated with a number of human diseases, including cancer and autoimmunity. Currently, it is unclear why some individuals with apparently proper responses to EBV develop associated diseases while others do not, but surely genetic and environmental factors, including life style and past pathogen encounters, play a role. In any case, a prophylactic EBV vaccine will be beneficial in preventing EBV-associated diseases. There is currently no pharmacological cure for EBV, and no preventative or therapeutic vaccinations are available. We designed a powerful multi-epitope peptide vaccine against EBV using in silico analysis. The vaccine is made up of 844 amino acids derived from three different types of proteins (Envelope, Capsid, EBNA) found in two different viral strains. These proteins have crucial functions in the infection of host cells and the control of the immune system of the host.

Multi-epitope vaccines require adjuvants because they increase the immunologic properties of vaccine structures. Previously, we used rOv-ASP-1, a recombinant Onchocerca volvulus activation associated protein-1, as an adjuvant and linked it to the vaccine’s N and C terminus (18).

The recombinant protein rOv-ASP 1 from Onchocerca volvulus may activate and mature naive human DCs, suggesting that it could be utilized as an innate adjuvant to produce balanced Th1 and Th2 responses to bystander vaccine immunogens. It also induced a Th1biased response with a few vaccine immunogens, as seen by significant induction of Th1associated IgG2a and IgG2b antibody responses and elevated production of Th1 cytokines such IL-2, IFN-γ, TNF-α, and IL-6 (26). To connect the adjuvant to N and C terminus of designed vaccine, we used EAAAK linker. Many recombinant fusion proteins have been constructed using alpha helix forming linkers with the sequence (EAAAK)n (27).

Many natural linkers have -helical structures, as George and Heringa predicted (28).

With intrasegment hydrogen bonding and a tightly packed backbone, the helical structure Was stiff and stable (29).

As a result, stiff -helical linkers might serve as rigid spacers between protein domains (30).

The KK linker was utilized to connect the different components of the vaccine in this investigation (31, 32). Cathepsin B, a lysosomal protease involved in the processing of immunogenic peptides for presentation on the cell surface in an MHC-II limited immunogen presentation, targets the Lysine linker. It also helps to reduce junctional immunogenicity by preventing the production of antibodies against the peptide sequence that can occur when separate epitopes are linked linearly (33). Immunogenicity is also boosted by KK linkers (34, 35).

An effective vaccine should have acceptable physicochemical characteristics throughout manufacture, formulation, storage, and consumption in addition to triggering immunological response. The proposed vaccine was stable, with a stability index of 33.57 and a pI of 10.10, according to bioinformatic predictions. Induction humoral responses rely heavily on conformational B-cell epitopes. The presence of a large number of B-cell epitopes in the vaccine molecule implies that this structure has a high capacity to activate B lymphocytes. To efficiently transport vaccine protein into immunogen-presenting cells, the vaccine must attach to immunological receptors (HLA class I histocompatibility immunogen, A-2 alpha chain, and HLA class II histocompatibility immunogen DQ alpha chain) in the correct way.

HLA class I immunogen is a kind of histocompatibility immunogen. In the presence of B2M/beta 2 microglobulin, immunogen-presenting cells show predominantly viral and tumor-derived peptides for identification by the alpha-beta T cell receptor (TCR) on HLA-A-restricted CD8-positive T cells. It guides the immunological response of immunogen-specific T cells to destroy infected or transformed cells (36).

Also, by displaying peptides generated from extracellular proteins, HLA-DQA1 plays a critical function in the immune system. Immunogen-presenting cells (APC: B) express class II molecules. Lymphocytes, dendritic cells, and macrophages are examples of these cells. Binds peptides generated from immunogens that enter the immunogen presentation cell’s (APC) endocytic pathway and displays them on the cell surface for CD4 T-cell identification (37). Docking analysis revealed that the vaccine protein binds correctly with immunological receptors. Also, it has a high-quality structure and suitable characteristics such as high stability.

The strategy that we followed to design the EBV vaccine relied on combining legacy experimentation consisting of experimentally defined epitopes with immunoinformatics predictions. The main advantage of this approach is that of saving time and resources as it mainly relies on experimentally validated epitopes, not on predicted epitopes, using immunoinformatics to identify those that are more suitable for epitope vaccine design.

This study has limitations that may handicap its translation into an EBV vaccine. Appropriate immunogen processing is a key limiting factor in the immunogenicity of T cell epitopes. Therefore, we selected experimental T cell epitopes that were shown to be processed and presented in the course of a natural infection with EBV and assumed that T cell epitope immunogenicity will be then only determined by their binding to MHC molecules.

## 5 Conclusion

The recent shift of interest from conventional to mRNA vaccines has proven its usefulness. The mRNA vaccine is able to induce a proper immune response towards the subject pathogen and provide long-lasting immunity. Despite no major advances to date, vaccinations remain a potential therapeutic strategy for suppressing the virulence of the Epstein-Barr virus, which is linked to infectious mononucleosis and various cancers in humans. In our study we have used an immunoinformatic and structural bioinformatics approach to rationally develop an mRNA vaccine for immune protection against EBV. The selection of candidate proteins and prioritization of epitopes were based on tested protocols. Our results demonstrated that the multi-epitope vaccine might be potentially immunogenic and induce humoral and cellular immune responses against EBV. This vaccine can interact appropriately with immunological receptors Also, it has a high-quality structure and suitable characteristics such as high stability.

## Data availability statement

The datasets presented in this study can be found in online repositories. The names of the repository/repositories and accession number(s) can be found in the article/supplementary material.

## Author contributions

All authors contributed to the article and approved the submitted version.
